# Hepato-Protective Effects of Delta-Tocotrienol and Alpha-Tocopherol in Patients with Non-Alcoholic Fatty Liver Disease: Regulation of Circulating MicroRNA Expression

**DOI:** 10.3390/ijms24010079

**Published:** 2022-12-21

**Authors:** Muhammad Amjad Pervez, Dilshad Ahmed Khan, Sayed Tanveer Abbas Gilani, Safia Fatima, Aamir Ijaz, Sumbal Nida

**Affiliations:** Armed Forces Institute of Pathology, National University of Medical Sciences, Rawalpindi 46000, Pakistan; dramjad14@gmail.com (M.A.P.); drtanveer2017@yahoo.com (S.T.A.G.); sofy_asim@hotmail.com (S.F.); ijaz_aamir@hotmail.com (A.I.); docsumbalnida@gmail.com (S.N.)

**Keywords:** δ-tocotrienol, α-tocopherol, circulating microRNAs, inflammation, apoptosis, non-alcoholic fatty liver disease

## Abstract

MicroRNAs (miRNAs) play a key role in the regulation of genes for normal metabolism in the liver. Dysregulation of miRNAs is involved in the development and progression of non-alcoholic fatty liver disease (NAFLD). We aimed to explore changes in circulating miRNA expression in response to delta-tocotrienol (δT3) and alpha-tocopherol (αTF) supplementation and correlate them with relevant biochemical markers in patients with NAFLD. In total, 100 patients with NAFLD were randomized to either receive δT3 (*n* = 50) 300 mg or αTF (*n* = 50) 268 mg twice/day for 48 weeks. Plasma expression of miRNA-122, -21, -103a-2, -421, -375 and -34a were determined at baseline, 24 and 48 weeks of intervention using RT-qPCR. Both δT3 and αTF significantly downregulated expression of miRNA-122, -21, -103a-2, -421, -375 and -34a. Moreover, δT3 was more effective than αTF in reducing expression of miRNA-375 and -34a. A significant correlation was observed between miRNA expression and biochemical markers of hepatic steatosis, insulin resistance (IR), oxidative stress (OS), inflammation and apoptosis. δT3 and αTF exert hepato-protective effects by downregulating miRNAs involved in hepatic steatosis, IR, OS, inflammation and apoptosis in patients with NAFLD. Furthermore, δT3 has more pronounced effects than αTF in reducing miR-375 and miR-34a, which are linked to regulation of inflammation and apoptosis.

## 1. Introduction

Non-alcoholic fatty liver disease (NAFLD) is defined as the presence of steatosis in >5% of hepatocytes, diagnosed either histologically or radiologically, in the absence of secondary causes of hepatic steatosis such as viral hepatitis, alcohol intake or hereditary liver diseases. NAFLD is a spectrum of liver diseases ranging from simple steatosis to non-alcoholic steatohepatitis (NASH), defined by steatosis with inflammation, hepatocyte injury and fibrosis, to cirrhosis and potentially hepatocellular carcinoma. Due to rapidly increasing prevalence of obesity and type 2 diabetes mellitus (T2DM), NAFLD has now become the most common cause of chronic liver disease worldwide, placing a significant burden on healthcare systems globally [1].

MicroRNAs (miRNAs or miRs) are small, non-coding endogenous RNA molecules that regulate target genes at the post-transcriptional level by binding to complementary sites in 3′-untranslated regions (3′-UTR) of mRNAs, causing translational repression and/or mRNA cleavage. miRNAs are secreted into extracellular fluids where they circulate in vesicles such as exosomes or bind to proteins and are thus protected from RNase-mediated degradation [2].

Several studies have demonstrated that the expression profile of circulating miRNAs in NAFLD patients is dysregulated compared with healthy controls. Moreover, the dysregulated expression pattern of circulating miRNAs mirrors the histological changes and the molecular events occurring in NAFLD. Thus, circulating miRNAs can serve as diagnostic biomarkers and therapeutic targets for the treatment of NAFLD [1,2,3,4].

The dysregulated miRNAs are involved in the pathogenesis of NAFLD, mainly through their effects on hepatic lipid metabolism, insulin resistance, oxidative stress, inflammation, apoptosis and fibrosis [3]. The sedentary lifestyle and consumption of an obesogenic diet result in deranged hepatic lipid metabolism and insulin resistance. Insulin resistance exacerbates lipid accumulation in the hepatocytes driven by increased lipid influx (from diet or adipose tissue lipolysis) and increased de novo lipogenesis as well as impaired lipid oxidation and export [1]. Notably, miR-122, miR-21 and miR-34a, identified as upregulated in NAFLD, play critical roles in deranged hepatic lipid metabolism and hepatic steatosis by regulating multiple hepatic pathways such as SREBP1-c, SIRT1 and PPARα [5]. Similarly, miR-103a-2, which is upregulated in NAFLD, induces insulin resistance by targeting caveolin-1 (Cav1), a critical regulator of the insulin receptor stability and insulin action [6].

The progression of NAFLD from simple steatosis to NASH is a result of lipotoxicity and the overproduction of reactive oxygen species (ROS). ROS lead to the recruitment of immune cells, the release of pro-inflammatory cytokines, chronic inflammation and liver damage through apoptosis and fibrosis. At these stages of NAFLD progression, miRNA-regulated pathways are also implicated [1]. For instance, miR-421, whose expression is increased in NAFLD, induces oxidative damage by inhibiting the SIRT3/FOXO3 pathway [7]. Similarly, the upregulated miR-375 participates in the inflammatory response [8], while miR-34a is involved in the induction of hepatocyte apoptosis in NAFLD [9].

Currently, there is no approved treatment for NAFLD. The current standard of care is lifestyle modification through weight loss, increased physical activity, and a low-calorie diet [10]. Therefore, recent studies have focused on the efficacy of nutritional supplements as a complementary therapy in the management of NAFLD.

Delta-tocotrienol (δT3) and alpha-tocopherol (αTF) are closely-related phytochemicals with proven metabolic health-promoting properties. We have recently reported the preventive and protective effects of δT3 and αTF supplementation on markers of hepatic steatosis (FLI and L/S ratio), insulin resistance (HOMA-IR), oxidative stress (MDA), inflammation (hs-CRP, TNF-α, IL-6, leptin and adiponectin) and hepatocyte apoptosis (CK18-M30) in patients with NAFLD [11]. In the current work, we aimed to explore changes in selected liver-related circulating miRNAs expression profiles in response to δT3 and αTF supplementation plus lifestyle modifications and correlate these changes with markers of hepatic steatosis (FLI and L/S ratio), insulin resistance (HOMA-IR), oxidative stress (MDA), inflammation (hs-CRP, TNF-α, IL-6, leptin and adiponectin) and apoptosis (CK18-M30) in patients with NAFLD.

## 2. Results

### 2.1. Patients’ Characteristics

**Figure 1:**
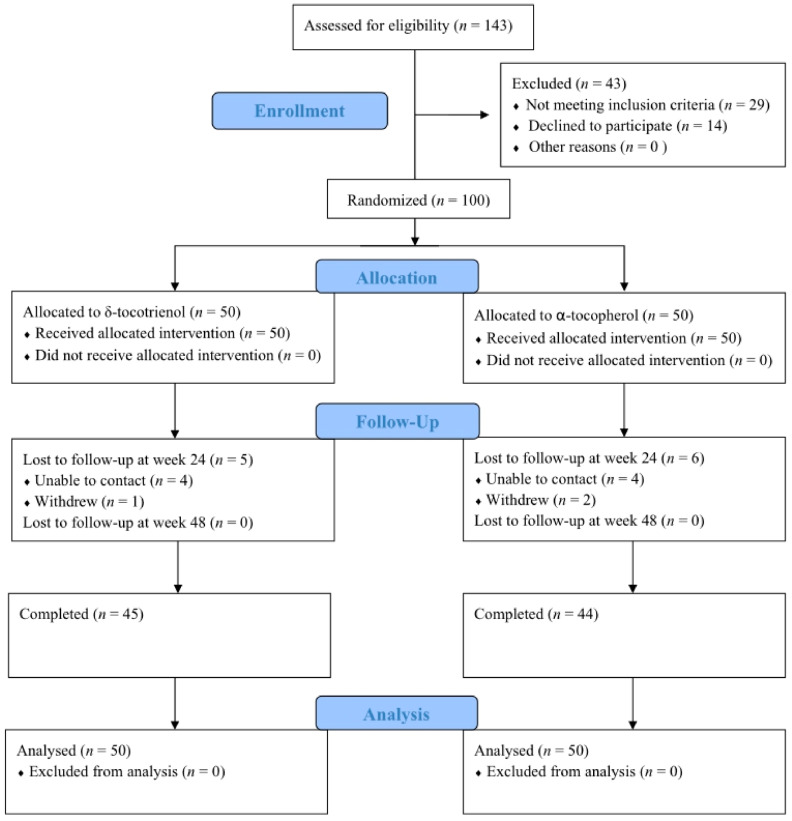
CONSORT flow diagram.

The study included a total of 100 patients (58 men and 42 women; mean age 47.7 years (range: 25–66 years)) with NAFLD that were randomized in 1:1 fashion (50 in each group). Of these, 89 patients, 45 (45%) in the δT3 and 44 (44%) in the αTF group, completed the study. However, intention-to-treat analysis was used, so data for all 100 patients were included in the final analysis (Figure 1). The baseline characteristics of both study groups are summarized in Table 1. There were no significant differences between the two groups in terms of demographic characteristics and miRNA expression at baseline.

### 2.2. Changes in Dietary Intake and Physical Activity

There were no significant differences between the two groups in terms of physical activity levels at baseline as well as at the end of study (*p* > 0.05).

There were no significant differences between the two groups in terms of dietary intake at baseline (*p* > 0.05). There were significant changes in dietary intake (*p* < 0.001) in both groups at 24 and 48 weeks of the study as compared to baseline. However, the between-group differences at 24 and 48 weeks were not significant (*p* > 0.05) (Table S2, Supplementary Materials).

### 2.3. Comparison of Mean Changes in miRNA Expression from Baseline to 24 and 48 Weeks

The mean ± SD changes in miRNAs expression from baseline to 24 and 48 weeks (∆∆Ct) of intervention are shown in Table 2. There were no significant differences (*p* > 0.05) in the ∆∆Ct values of miR-122, miR-21, miR-103a-2 and miR-421 between the two groups at 24 and 48 weeks of intervention. However, there were significant differences (*p* < 0.05) in the ∆∆Ct values of miR-375 and miR-34a between the two groups at 24 and 48 weeks of intervention.

### 2.4. Comparison of Fold Changes in miRNA Expression at 24 and 48 Weeks

The median (inter-quartile range) fold change (FC) in miRNA expression level from baseline to 24 and 48 weeks of intervention are shown in Table 3. There was a >2-fold downregulation in the expression levels of the tested miRNAs in both δT3 and αTF group at 24 and 48 weeks of intervention as compared to baseline.

For between-group comparisons, there were no significant differences (*p* > 0.05) in the downregulation of miR-122, miR-21, miR-103a-2 and miR-421 at 24 and 48 weeks of the intervention. However, there were significant differences (*p* < 0.05) in the downregulation of miR-375 and miR-34a, with the δT3 group exhibiting a significantly greater down-regulation in expression levels of miR-375 and miR-34a as compared to αTF group at both 24 and 48 weeks of intervention.

### 2.5. Correlation between miRNAs and Biochemical Markers

As the expression levels of all the tested miRNAs were downregulated significantly during the course of intervention, correlation analyses between the miRNA expression and the corresponding values of the relevant biochemical markers were conducted. ΔCt values were linearized using 2^−(ΔCT)^, which were subsequently used to test the correlation between miRNA expression and biochemical markers. The correlation analyses revealed significant correlations between miRNA expression levels and the relevant biochemical markers at baseline, 24 and 48 weeks of the intervention (Table 4).

## 3. Discussion

In this work, we investigated the effects of δT3 and αTF supplementation on modulation of plasma miRNA expression in patients with NAFLD. We found a significant downregulation (>2-fold; *p* < 0.05) in the expression of all the tested miRNAs during the course of intervention as compared to baseline. On comparison of the two groups, there were no significant differences (*p* > 0.05) in the downregulation of miR-122, miR-21, miR-103a-2 and miR-421. However, the downregulation of miR-375 and miR-34a was significantly greater (*p* < 0.05) in the δT3 group as compared to αTF at both assessment points. Just like improvement in biochemical markers as previously reported [11], the maximum effect of supplementation on miRNA expression also occurred during the first 24 weeks and was sustained throughout the study period. Moreover, we observed a significant correlation between selected miRNA levels and the relevant biochemical parameters at each of the three assessment points.

In the present study, we observed a significant downregulation in the circulating levels of miR-122 and miR-21 from pre- to postintervention. Also, the expression of both these miRNAs significantly correlated with FLI and L/S ratio at baseline. Moreover, a decrease in their expression correlated with the changes in FLI and L/S ratio from pre- to postintervention. This significant correlation indicates that the improvement in hepatic steatosis as seen in the current study may be mediated by downregulation of miR-122 and miR-21 expression.

The decrease in the levels of miR-122 along with an amelioration of hepatic steatosis as observed in the current study is consistent with several previous reports [12,13,14,15].

Regarding the mechanism of steatosis reduction following miR-122 downregulation, administration of antisense oligonucleotides (ASO)—which inhibit miR-122 function—resulted in increased hepatic fatty acid oxidation and a significant improvement in liver steatosis in diet-induced obese mice. These effects were accompanied by a downregulation of mRNA levels of various lipogenic genes (SREBP1, FASN) and an upregulation of hepatic fatty-acid oxidation mechanisms such as increased levels of activated AMPKa1 [16,17]. Moreover, it has been reported that downregulation of miR-21 expression results in decreased hepatic steatosis through upregulation of activities of PTEN [18], HBP1-p53 [19] and PPARα [20] pathways. Activation of these pathways decreases hepatic steatosis by downregulating the expression of SREBP1c and other lipogenic genes and by enhancing fatty acid oxidation. 

In the present study, we found a significant downregulation in the expression of miR-103a-2 from pre- to postintervention. Moreover, because the change in miR-103a-2 levels was shown to correlate with the change in insulin level and HOMA-IR in our study, the decreased miR-103a-2 levels after the intervention may be involved in the underlying mechanisms of an improved glucose metabolism and insulin sensitivity.

The reduction in the levels of miR-103a-2 along with an amelioration of marker of insulin resistance as observed in the present study is consistent with a previous report [21].

Mechanistically, miR-103a directly targets caveolin-1 (Cav1), which is a critical regulator of insulin receptor stability. Inhibition of miR-103a increased the expression of caveolin-1 in adipocytes which resulted in a parallel increase in insulin receptor stability and enhanced insulin sensitivity. In contrast, gain of miR-103a function in either liver or adipocytes was sufficient to induce insulin resistance by downregulating caveolin-1 [6].

In the present study, we observed a significant downregulation in circulating miR-421 from pre- to postintervention. The downregulation of this miRNA may be involved in reduced oxidative damage after the intervention. The reasoning is supported by the significant correlation between miR-421 and MDA observed at baseline and during the intervention.

Mechanistically, SIRT3 is a target of miR-421. Inhibition of miR-421 by antisense oligonucleotide resulted in upregulation of SIRT3, a FOXO3 activator. SIRT3-mediated deacetylation of FOXO3 induces the expression of antioxidant enzymes MnSOD and catalase, which reduce the levels of cellular ROS, thereby protecting cells from oxidative damage and reducing MDA levels [7].

In the present study, we observed a significant downregulation in the expression of circulating miR-375 from pre- to postintervention. Notably, the magnitude of downregulation was significantly greater in the δT3 group as compared to αTF group. Consistent with this downregulation in miR-375 expression, there was a significantly greater reduction in the serum IL-6, TNF-a and leptin and a significantly greater increase in serum adiponectin in the δT3 group compared to αTF group, as previously reported [11]. Moreover, we observed a significant correlation between miR-375 relative expression and these inflammation-related markers at baseline as well as during the course of intervention. These results suggest that the downregulation of miR-375 expression postintervention may be involved in the underlying mechanisms of improved inflammatory profile in the present study.

Mechanistically, adiponectin receptor 2 (AdipoR2) is a direct target gene of miR-375 and the downregulation/inhibition of miR-375 leads to an increased AdipoR2 expression. In palmitic acid (PA)-treated human hepatocellular carcinoma HepG2 cells, the downregulation of miR-375 expression significantly reduced IL-6, TNF-α, and leptin, and increased adiponectin levels. Moreover, the reduction of IL-6, TNF-α and leptin as well as the increases of adiponectin by miR-375 inhibition were reversed by the silencing of AdipoR2. These results indicate that inhibition of miR-375 regulates IL-6, TNF-α, leptin and adiponectin, at least partly, through upregulating the expression of AdipoR2 in PA-treated HepG2 cells [8].

The results of the present study demonstrated a significant decrease in the plasma expression of miR-34a from pre- to postintervention. Notably, the decrease in the expression of this miRNA was also significantly greater in the δT3 group as compared to αTF. This observation is consistent with the finding of a significantly greater reduction in CK-18 levels in the δT3 group compared to αTF as previously reported [11]. Collectively, these findings point towards a critical role of decreased miR-34a expression in reducing hepatocyte apoptosis in the current study. This role is further supported by the presence of a significant correlation between miR-34a and CK-18 levels at baseline as well as when assessing the correlations over time.

The reduction in the levels of miR-34a along with an amelioration of markers of hepatic apoptosis as observed in the current study is consistent with a previous report. Mechanistically, the best characterized direct target of miR-34a is SIRT1, a NAD-dependent deacetylase that modulates hepatic apoptosis. miR-34a suppression-mediated increase in SIRT1 and subsequent decrease in p53 acetylation and transcription results in inhibition of pro-apoptotic genes, such as PUMA, and finally, apoptosis [9].

### Study Limitations

This study has a few limitations. We did not evaluate mRNA and protein expression of the predicted target genes of the tested miRNAs, and there was loss to follow-up of some patients due to COVID-19. Additionally, as it was a single-center study with a small sample size, the generalizability of the findings may be limited.

## 4. Methods and Materials

### 4.1. Study Design and Setting

In the present study, plasma samples obtained in a randomized, double-blinded, active-controlled clinical trial were used to explore changes in selected miRNA expression profiles in response to δT3 and αTF supplementation. The study was conducted at the Armed Forces Institute of Pathology (AFIP), National University of Medical Sciences, Rawalpindi, Pakistan. The study conformed with guidelines of the Declaration of Helsinki (2013). The study protocol was approved by the Institutional Review Board of AFIP and was registered with the Sri Lankan Clinical Trial Registry (https://slctr.lk/SLCTR/2019/038, accessed on 6 October 2022).

### 4.2. Patients

Patients were included after informed consent and following the inclusion criteria, consisting of: (1) men and women aged 20–70 years, (2) FLI of ≥60, (3) L/S ratio of <1.1, and (4) ALT within reference range or mildly raised. Exclusion criteria comprised chronic viral hepatitis, alcoholic liver disease, autoimmune liver disease, hemochromatosis, Wilson’s disease, all systemic diseases (including diabetes mellitus and liver cirrhosis) and use of nutritional/vitamin supplements.

### 4.3. Randomization and Intervention

NAFLD patients (*n* = 100) were randomized by hospital pharmacist to either receive δT3 (*n* = 50) 300 mg or αTF (*n* = 50) 268 mg (400 IU) twice daily for 48 weeks. Both the patients and investigators were blinded to treatment allocation. Patients were advised to consume one capsule in morning after breakfast and the other at night after dinner. Patients were also advised to engage in lifestyle changes, i.e., diet and physical activity [10]. The patients were regularly monitored for compliance to the study protocol, and any adverse effects of supplements by using telephone calls and regular follow-up visits.

### 4.4. Assessment of Dietary Intake and Physical Activity

Assessment of dietary intake was performed at baseline, 24 and 48 weeks of intervention by using 24 h dietary recall for 3 days (2 weekdays and 1 weekend) and analyzed with Nutritionist-4 software. The physical activity was assessed using the International Physical Activity Questionnaire (IPAQ) [22].

### 4.5. Selection of Targets miRNAs

We carried out a comprehensive literature search and chose a panel of six miRNAs that were the most representative of each of the key processes found to be dysregulated in NAFLD [1,2,3,4]. The selected miRNAs included hsa-miR-122-5p (miR-122), hsa-miR-21-5p (miR-21), hsa-miR-103a-2-5p (miR-103a-2), hsa-miR-421 (miR-421), hsa-miR-375-5p (miR-375) and hsa-miR-34a-5p (miR-34a). The sequences of these miRNAs used to design primer assays are given in Table S1 (Supplementary Materials).

### 4.6. Quantification of miRNAs

#### 4.6.1. Blood Collection and Storage

Blood (5 mL) was drawn in EDTA tubes and plasma was separated within 1 h by centrifugation at 4000 rpm and 4 °C for 10 min. Then, 250 μL plasma was immediately homogenized in 750 μL TRIzol^™^ LS Reagent (Invitrogen Life Technologies, Carlsbad, CA, USA), frozen and stored at −80 °C until RNA extraction.

#### 4.6.2. RNA Extraction

Prior to RNA extraction, the frozen lysates were incubated at 37 °C in water bath until samples were completely thawed and salts were dissolved. Total RNA, including small RNA, was extracted using the Trizol LS method [23]. During extraction, 3.5 µL of C. elegans miRNA-39-3p (cel-miR-39-3p) (Qiagen) were spiked into each sample as normalizer.

Concentration (ng/μL) and purity of extracted RNA was assessed by measuring the absorbance at 260 nm (A260) and 280 nm (A280) using a NanoDrop ND-1000 spectrophotometer (Nanodrop Technologies, Wilmington, DE, USA). A A260/A280 ratio of 1.8–2.1 was considered as acceptable purity for downstream applications. Extracted RNA was stored at −80 °C.

#### 4.6.3. Quantitative Real-Time PCR (RT-qPCR)

Reverse transcription was carried out to generate single-stranded complementary DNA (cDNA) from total RNA using miRNA All-In-One cDNA Synthesis Kit (Applied Biological Materials Inc. Richmond, BC, Canada). Briefly, total RNA (300 ng/sample) was mixed with 2X miRNA cDNA Synthesis SuperMix (10 μL), 0.5 U/reaction Enzyme Mix (2 μL) and nuclease-free water to a final volume of 20 μL. The reaction mixtures were then incubated at 37 °C for 30 min, at 42 °C for 15 min and at 70 °C for 10 min. cDNA samples were stored at −20 °C. cDNA was amplified by PCR using BrightGreen-miRNA Mastermix and primers sets for each miRNA (Applied Biological Materials Inc. Richmond, BC, Canada). Briefly, 2.5 μL of cDNA was mixed with 1X BrightGreen miRNA qPCR MasterMix (10 μL) 300 nM forward primer (0.6 μL), 300 nM reverse primer (0.6 μL) and nuclease-free water (6.3 μL) to a final volume of 20 μL. Amplifications were performed in the Rotor-Gene™ Q real-time PCR cycler (Qiagen) with the following cycling conditions: 95 °C for 10 min, 40 cycles of 95 °C for 10 s, 63 °C for 15 s followed by incubation at 72 °C for 10 s. Amplifications were performed in triplicate for each RNA sample and cycle threshold (Ct) values were determined. The no-template was used as negative control in each PCR run. All Ct values higher than 35 were excluded.

A melting curve analysis was performed after the thermal profile to ensure specificity in the amplification.

Average Ct values of each target miRNA at baseline, 24 and 48 weeks were normalized to the corresponding average Ct values of spike-in cel-miR-39-3p and presented as ∆Ct = [Ct _(target miR)_ − Ct _(cel-miR-39−3p)_]. The change in each miRNA expression from baseline to 24 and 48 weeks was calculated as:

ΔΔCt = 24(48)-week ∆Ct [Ct _(target miRNA)_ − Ct _(cel-miR-39-3p)_] − Baseline ∆Ct [Ct _(target miRNA)_ − Ct _(cel-miR-39-3p)_]

Finally, the 2^−(ΔΔCt)^ method was used to calculate the fold change (FC) in each miRNA expression within the δT3 and αTF groups relative to the baseline.

Gene expression is significantly upregulated when FC is >2.0 and is reported as such. Gene expression is significantly downregulated when FC is <0.5. In such cases, taking negative inverse of FC, i.e., −1/FC, provides the fold reduction in the gene expression [24].

### 4.7. Sample Size Calculation

The sample size was calculated as described previously [11].

### 4.8. Statistical Analysis

Normality of distribution for quantitative data was checked using Kolmogorov–Smirnov test. Results are presented as mean ± standard deviation (SD) or as medians (inter-quartile range) for continuous variables and frequencies and percentages for categorical variables. The baseline data were compared using independent *t*-test or chi-square test as appropriate.

To control for the confounding effects of changes in dietary intakes on miRNA expression levels, we used ANCOVA model for each miRNA. The model consisted of the between-subjects factor as the grouping variable (with δT3 and αTF as levels), the baseline miRNA expression (ΔCt) and the changes in energy intake from baseline to 24 and 48 weeks as covariates, and the changes in miRNA expression from baseline to 24 and 48 weeks (∆∆Ct) as dependent variables. The estimated between-group differences which represent the treatment effect (mean difference = MD) are reported with 95% confidence intervals (CIs) and p values. Mann–Whitney U test was used to assess differences in miRNA fold changes between the two groups at 24 and 48 weeks. Spearman’s correlation coefficients were calculated to examine the relationship between a specific miRNA level and the relevant biochemical parameter. Statistical analysis was conducted using SPSS version 21 for Windows (IBM Corp. Armonk, NY, USA) and differences/correlations with two-tailed *p* < 0.05 were considered significant.

## 5. Conclusions

This study demonstrated that daily supplementation of δT3 or αTF, along with lifestyle changes, exerts hepato-protective effects by downregulating miRNAs involved in hepatic steatosis, insulin resistance, oxidative stress, inflammation and apoptosis in patients with NAFLD. Moreover, δT3 exerts a more pronounced effect than αTF in reducing miR-375 and miR-34a, which are linked to the regulation of inflammation and apoptosis, respectively. Further studies illustrating miRNA–mRNA–protein interactions and regulatory pathways are warranted.

## Figures and Tables

**Table 1:** Baseline characteristics of patients in each group.

Parameter	δT3 (*n* = 50)	αTF (*n* = 50)	† *p*-Value
Sex			
Female	23 (46.0) ‡	19 (38.0)	0.418
Male	27 (54.0)	31 (62.0)	
Age (years)	47.28 ± 8.36 §	48.04 ± 7.66	0.637
hsa-miR-122-5p (∆Ct)	2.77 ± 1.51	3.42 ± 2.26	0.194
hsa-miR-21-5p (∆Ct)	2.42 ± 1.65	2.97 ± 2.44	0.195
hsa-miR-103a-2-5p (∆Ct)	2.42 ± 2.08	2.88 ± 2.45	0.306
hsa-miR-421 (∆Ct)	2.67 ± 1.77	2.83 ± 2.31	0.699
hsa-miR-375-5p (∆Ct)	2.61 ± 1.73	2.74 ± 2.33	0.742
hsa-miR-34a-5p (∆Ct)	2.65 ± 1.70	2.74 ± 2.31	0.83
Physical activity level			
Light	44 (88.0)	42 (84.0)	
Moderate	6 (12.0)	8 (16.0)	0.774
Vigorous	0 (0.0)	0 (0.0)	

δT3: δ-tocotrienol; αTF: α-tocopherol; hsa, Homo sapiens; miR: microRNA; Ct: cycle threshold; ∆Ct: Ct miR minus Ct miR-Cel-39-3p. † Between groups by independent *t*-test, chi-square test or Fisher’s exact test as appropriate, ‡ Values are frequency with percentage, § Values are mean ± standard deviation (SD).

**Table 2:** Mean changes in miRNA expression from baseline to 24 and 48 weeks.

	∆∆Ct			
miRNA	δT3 (*n* = 50)	αTF (*n* = 50)	† MD (95% CI)	‡ *p*-Value
hsa-miR-122-5p				
Week 24	1.30 ± 0.93 §	1.17 ± 0.72	0.24 (−0.05, 0.60)	0.093
Week 48	1.46 ± 0.95	1.36 ± 0.75	0.27 (−0.08, 0.57)	0.138
hsa-miR-21-5p				
Week 24	1.21 ± 0.56	1.20 ± 0.71	0.11 (−0.11, 0.34)	0.317
Week 48	1.36 ± 0.64	1.33 ± 0.73	0.13 (−0.12, 0.38)	0.316
hsa-miR-103a-2-5p				
Week 24	1.24 ± 0.57	1.19 ± 0.61	0.06 (−0.17, 0.29)	0.613
Week 48	1.37 ± 0.61	1.36 ± 0.65	0.01 (−0.23, 0.26)	0.804
hsa-miR-421				
Week 24	1.22 ± 0.50	1.11 ± 0.44	0.15 (−0.02, 0.32)	0.086
Week 48	1.41 ± 0.55	1.32 ± 0.51	0.12 (−0.08, 0.32)	0.227
hsa-miR-375-5p				
Week 24	1.28 ± 0.55	1.07 ± 0.47	0.22 (0.03, 0.42)	0.028
Week 48	1.48 ± 0.65	1.23 ± 0.53	0.25 (0.03, 0.48)	0.03
hsa-miR-34a-5p				
Week 24	1.29 ± 0.52	1.03 ± 0.44	0.29 (0.11, 0.47)	0.002
Week 48	1.41 ± 0.56	1.21 ± 0.54	0.23 (0.02, 0.43)	0.029

δT3: δ-tocotrienol; αTF: α-tocopherol; MD: mean difference; hsa, Homo sapiens; miRNA/miR: microRNA; Ct: cycle threshold. ∆∆Ct: ∆Ct at 24(48) weeks minus ∆Ct at baseline. † δT3 minus αTF group. ‡ Between-group by analysis of covariance adjusted for baseline values and mean changes of daily energy intake. § Values are mean ± standard deviation (SD) change.

**Table 3:** Fold changes in microRNA expression from baseline to 24 and 48 weeks of intervention.

miRNA	δT3 (*n* = 50)		αTF (*n* = 50)		† *p*-Value
	FC	–1/FC	FC	–1/FC	
hsa-miR-122-5p					
Week 24	0.41 (0.27, 0.59) ‡	–2.42	0.44 (0.33, 0.58)	–2.22	0.482
Week 48	0.36 (0.23, 0.55)	–2.73	0.38 (0.30, 0.52)	–2.60	0.636
hsa-miR-21-5p					
Week 24	0.42 (0.33, 0.49)	–2.39	0.46 (0.29, 0.55)	–2.16	0.685
Week 48	0.36 (0.29, 0.46)	–2.73	0.40 (0.27, 0.49)	–2.49	0.768
hsa-miR-103a-2-5p					
Week 24	0.39 (0.34, 0.53)	–2.55	0.41 (0.36, 0.48)	–2.43	0.352
Week 48	0.35 (0.30, 0.47)	–2.82	0.36 (0.31, 0.42)	–2.77	0.736
hsa-miR-421					
Week 24	0.41 (0.33, 0.47)	–2.42	0.43 (0.39, 0.46)	–2.32	0.131
Week 48	0.36 (0.28, 0.41)	–2.77	0.37 (0.33, 0.40)	–2.72	0.423
hsa-miR-375-5p					
Week 24	0.39 (0.31, 0.48)	–2.54	0.45 (0.39, 0.52)	–2.18	0.012
Week 48	0.34 (0.26, 0.43)	–2.88	0.40 (0.34, 0.46)	–2.47	0.008
hsa-miR-34a-5p					
Week 24	0.40 (0.32, 0.45)	–2.48	0.43 (0.39, 0.56)	–2.27	0.002
Week 48	0.35 (0.30, 0.40)	–2.80	0.38 (0.32, 0.49)	–2.63	0.040

δT3: δ-tocotrienol; αTF: α-tocopherol; hsa, Homo sapiens; miRNA/miR: microRNA; FC: Fold change. Gene expression is recognized as downregulated when FC is <1. Taking negative inverse of FC (−1/FC) provides with fold reduction in expression. † Based on Mann–Whitney U test between groups. ‡ Values are median with inter-quartile range (IQR).

**Table 4:** Correlation between microRNAs and relevant biochemical markers.

	hsa-miR-122		hsa-miR-21		hsa-miR-103a-2		hsa-miR-375		hsa-miR-421		hsa-miR-34a	
	r	*p*	r	*p*	r	*p*	r	*p*	r	*p*	r	*p*
FLI												
Week 0	0.75	<0.001	0.81	<0.001							0.78	<0.001
Week 24	0.8	<0.001	0.83	<0.001							0.81	<0.001
Week 48	0.81	<0.001	0.79	<0.001							0.79	<0.001
L/S ratio												
Week 0	−0.60	<0.001	−0.65	<0.001							−0.67	<0.001
Week 24	−0.58	<0.001	−0.60	<0.001							−0.62	<0.001
Week 48	−0.56	<0.001	−0.59	<0.001							−0.60	<0.001
HOMA-IR												
Week 0					0.84	<0.001						
Week 24					0.76	<0.001						
Week 48					0.73	<0.001						
hs-CRP (mg/L)												
Week 0							0.83	<0.001				
Week 24							0.79	<0.001				
Week 48							0.77	<0.001				
IL-6 (pg/mL)												
Week 0							0.66	<0.001				
Week 24							0.59	<0.001				
Week 48							0.58	<0.001				
TNF-α (pg/mL)												
Week 0							0.7	<0.001				
Week 24							0.65	<0.001				
Week 48							0.51	<0.001				
Leptin (ng/mL)												
Week 0							0.7	<0.001				
Week 24							0.73	<0.001				
Week 48							0.72	<0.001				
AdpN (µg/mL)												
Week 0							−0.55	<0.001				
Week 24							−0.52	<0.001				
Week 48							−0.50	<0.001				
MDA (ng/mL)												
Week 0									0.6	<0.001	0.59	<0.001
Week 24									0.52	<0.001	0.48	<0.001
Week 48									0.47	<0.001	0.45	<0.001
CK18-M30 (mIU/mL)												
Week 0											0.56	<0.001
Week 24											0.5	<0.001
Week 48											0.41	<0.001

hsa, Homo sapiens; miR: microRNA; FLI: fatty liver index; L/S ratio: liver to spleen attenuation ratio; HOMA-IR: Homeostatic Model Assessment of Insulin Resistance; hs-CRP: high-sensitive C-reactive protein; IL-6: interleukin-6; TNF-α: tumor necrosis factor alpha; AdpN: adiponectin; MDA: malondialdehyde; CK18-M30: Cytokeratin18-M30. Correlation analysis was performed by Spearman’s rank test.

## Data Availability

The data that support the findings of this study are available from the corresponding author upon reasonable request.

## Supplementary Materials

The following supporting information can be downloaded at: https://www.mdpi.com/1422-0067/24/1/79/s1.

## Abbreviations

RT-qPCR: reverse transcription quantitative polymerase chain reaction; FLI: fatty liver index; L/S ratio: liver-to-spleen attenuation ratio on CT scan; HOMA-IR: homeostatic model assessment for insulin resistance; MDA: malondialdehyde; hs-CRP: high sensitivity C-reactive protein; TNF-α: tumor necrosis factor-alpha; IL-6: interleukin-6; CK18-M30: cytokeratin 18-M30; ALT: alanine transaminase; EDTA: ethylenediamine tetra-acetic acid; SREBP-1c: sterol regulatory element-binding protein 1c; FASN: fatty acid synthase; AMPKa1: AMP-activated protein kinase alpha 1; PTEN: phosphatase and tensin homolog; HBP1: HMG box protein 1; p53: tumor protein P53; PPARα: peroxisome proliferator-activated receptor alpha; SIRT1,3: sirtuin 1,3; FOXO3: forkhead box O3; MnSOD: manganese superoxide dismutase; PUMA: p53 upregulated modulator of apoptosis.
