# Supplementary material for: Hepato-Protective Effects of Delta-Tocotrienol and Alpha-Tocopherol in Patients with Non-Alcoholic Fatty Liver Disease: Regulation of Circulating MicroRNA Expression
Source: Int J Mol Sci. 2022 Dec 21;24(1):79. doi: 10.3390/ijms24010079 (PMC9820400; doi:10.3390/ijms24010079)
Supplement: Supplementary file 1 [file ijms-24-00079-s001.zip › ijms-1986257-supplementary.pdf]

Table S1: Sequences of tested miRNAs and spike-in reference used by the manufacturer to design primers for RT-qPCR

**miRNA/gene**	**miRBase Accession**	**Mature miRNA sequence**
hsa-mir-122-5p	MIMAT0000421	5’ UGGAGUGUGACAAUGGUGUUUG ’3
hsa-mir-21-5p	MIMAT0000076	5’ UAGCUUAUCAGACUGAUGUUGA ’3
hsa-mir-103a-2-5p	MIMAT0009196	5’ AGCUUCUUUACAGUGCUGCCUUG ’3
hsa-mir-421	MIMAT0003339	AUCAACAGACAUUAAUUGGGCGC
hsa-mir-375-5p	MIMAT0037313	5’ GCGACGAGCCCCUCGCACAAACC ’3
hsa-mir-34a-5p	MIMAT0000255	5’ UGGCAGUGUCUUAGCUGGUUGU ’3
cel-miR-39-3p	MIMAT0000010	5’ UCACCGGGUGUAAAUCAGCUUG ’3

Table S2: Daily dietary intakes of the study participants at baseline, 24- and 48 weeks

Parameter	δT3 (n=50)	αTF (n = 50)	*p-value*
Energy (kcal/day)			
Baseline	2425.6 ± 340.2[†]	2336.9 ± 347.4	0.200[‡]
Week 24	2353.6 ± 311.1	2250.4 ± 309.5	0.088[§]
Week 48	2313.6 ± 293.2	2209.3 ± 292.2	0.094[§]
[¶]p-value	< 0.001	< 0.001	
Carbohydrate (g/day)			
Baseline	333.1 ± 44.7	323.8 ± 47.1	0.320[‡]
Week 24	322.3 ± 41.5	310.5 ± 44.3	0.166[§]
Week 48	317.1 ± 40.2	304.2 ± 42.4	0.105[§]
[¶]p-value	< 0.001	< 0.001	
Protein (g/day)			
Baseline	97.8 ± 17.3	92.9 ± 17.2	0.160[‡]
Week 24	103.0 ± 15.6	97.4 ± 14.8	0.090[§]
Week 48	105.1 ± 13.7	100.2 ± 12.9	0.147[§]
[¶]p-value	< 0.001	< 0.001	
Total fat (g/day)			
Baseline	78.1 ± 14.0	74.4 ± 12.7	0.182[‡]
Week 24	72.5 ± 13.5	68.8 ± 12.8	0.636[§]
Week 48	69.4 ± 13.1	65.7 ± 12.7	0.601[§]
[¶]p-value	< 0.001	< 0.001	

Abbreviations: δT3: δ-tocotrienol; αTF: α-tocopherol.

[†] Values are mean ± standard deviation (SD)
[‡] Between-groups by independent sample t-test
[§] Between-groups by analysis of covariance adjusted for baseline values
[¶] Within group by repeated measures analysis of variance
